# 

**Published:** 1838-04-11

**Authors:** 


☞ *Complaints having reached us, from several quarters, of the occasional miscarriage of this Journal, we can only say, that each subscriber's number is mailed under the supervision of a careful individual, and by him delivered at the Post-Office of this city. As the number of copies printed will, we fear, fall short of the demand, it will be impossible for us to supply the deficiencies in future.*

